# Exploiting MEK Inhibitor-Mediated Activation of ERα for Therapeutic Intervention in ER-Positive Ovarian Carcinoma

**DOI:** 10.1371/journal.pone.0054103

**Published:** 2013-02-04

**Authors:** June Y. Hou, Alicia Rodriguez-Gabin, Leleesha Samaweera, Rachel Hazan, Gary L. Goldberg, Susan Band Horwitz, Hayley M. McDaid

**Affiliations:** 1 Division of Gynecologic Oncology, Department of Obstetrics and Gynecology and Women’s Health, Montefiore Medical Center, Albert Einstein College of Medicine, Bronx, New York, United States of America; 2 Department of Molecular Pharmacology, Albert Einstein College of Medicine, Bronx, New York, United States of America; 3 Department of Pathology, Albert Einstein College of Medicine, Bronx, New York, United States of America; 4 Department of Medicine (Oncology), Albert Einstein College of Medicine, Bronx, New York, United States of America; Rutgers University, United States of America

## Abstract

While the clinical benefit of MEK inhibitor (MEKi)-based therapy is well established in *Raf* mutant malignancies, its utility as a suppressor of hyperactive MAPK signaling in the absence of mutated *Raf* or *Ras,* is an area of ongoing research. MAPK activation is associated with loss of ERα expression and hormonal resistance in numerous malignancies. Herein, we demonstrate that MEKi induces a feedback response that results in ERα overexpression, phosphorylation and transcriptional activation of ER-regulated genes. Mechanistically, MEKi-mediated ERα overexpression is largely independent of erbB2 and AKT feedback activation, but is ERK-dependent. We subsequently exploit this phenomenon therapeutically by combining the ER-antagonist, fulvestrant with MEKi. This results in synergistic suppression of tumor growth, in *vitro* and potentiation of single agent activity in *vivo* in nude mice bearing xenografts. Thus, we demonstrate that exploiting adaptive feedback after MEKi can be used to sensitize ERα-positive tumors to hormonal therapy, and propose that this strategy may have broader clinical utility in ERα-positive ovarian carcinoma.

## Introduction

Epithelial ovarian cancer (EOC), the most common type of ovarian cancer, is the fifth leading cause of female cancer mortality in the United States. Of the estimated 21,990 cases that occurred in 2011, more than two-thirds will die from the disease due to innate, or acquired drug resistance [1]. Recent insight into the pathogenesis of EOC suggests two distinct categories of tumorigenesis, designated type I and II [2]. Type I carcinoma include histologic subtypes such as low-grade serous, mucinous, endometrioid, and clear-cell. These tumors commonly afflict younger patients, have a low proliferative index, and an overall improved prognosis when compared to type II cancers that include the more common high grade serous neoplasms [3], [4]. Following an indolent course, up to 50% of type I patients will succumb to metastatic disease. Chemotherapeutic resistance associated with either type I or II EOC presents a therapeutic dilemma for many clinicians. Thus, the identification of mechanisms of resistance and subsequent development of alternate therapies is vital to patient outcome.

The Mitogen-Activated Protein Kinase (MAPK) signaling pathway is a major regulator of cell proliferation, survival and differentiation. Hyperactivation of this pathway occurs in EOC via gain of function mutations in *Ras* or *Raf*, (primarily in borderline, as well as type I ovarian carcinomas), which is thought to promote neoplastic transformation from low grade ovarian tumors to invasive type I disease [5], [6]. In addition, mutations in PTEN and PI3KCA contribute to the unique molecular signature of type I ovarian cancer. In contrast, type II cancers almost invariably involve p53 (TP53) mutations [7].

It is known that signaling networks such as MAPK interact with hormonal mediators, such as estrogen receptor alpha (ERα) in a non-genomic, estrogen (ES)-independent manner in hormonally-dependent malignancies [8]. ERα is expressed in 40–60% of EOC (protein and mRNA, respectively) and 50% of borderline ovarian cancers [9], [10]. Previous studies have identified several kinases, including components of the MAPK cascade that phosphorylate residues on ERα leading to transcription of ES-dependent target genes [11]. Additional studies have also established an inverse relationship between MAPK signaling and ERα genomic activity [12], [13]. What remains unclear is whether ERα expression confers tumor growth dependency on ES, and whether targeting ERα will modulate ovarian cancer cell growth or survival [14], [15].

The response rate to anti-estrogen therapies (AET) in clinical trials for patients with recurrent ovarian cancer ranges from 8%–17%, irrespective of ERα status [16]–[20]. Similar to breast cancer, where up to 50% of ERα-positive tumors are hormonally resistant de-novo, these trials in ovarian cancer suggest that inhibiting estrogen signaling on a receptor level has some efficacy; however it is not enough to produce a strong clinical response.

Blocking oncogenic *Ras* retards cell growth by causing cell cycle arrest and/or apoptosis, and *in vivo* models have demonstrated varying degrees of response to MEK inhibitors (MEKi) in tumor models [21]–[23], including endometrial cancer [21]. Currently, *Ras*-mutant malignancies, such as type I ovarian cancer, constitute a tumor class with unmet clinical need. Several MEKi’s are being developed in multiple cancer trials (http://clinicaltrials.gov). Given the interactions between MAPK signaling and ERα in ovarian cancer, we hypothesized that deregulation of MAPK modulates the intrinsic activity of ERα, and contributes to endocrine resistance in EOC. Thus, inhibition of the pathway using a selective MEKi may sensitize defined cohorts of ovarian cancer patients with ERα-positive disease to anti-estrogen therapy.

## Materials and Methods

### Cell Culture, Antibodies and Reagents

Cells were purchased from American Type Culture Collection (ATCC), or the NCI tumor repository and cultured in RPMI 1640 at 37°C in a humidified incubator with 5% CO_2_. All cell lines were cultured in medium supplemented with 10% fetal bovine serum (FBS), and cells with less than eight passages were used for all experiments. Antibodies used were from Cell Signaling Technologies except ERα (HC-20, Santa Cruz Biotechnology Inc., SC-543). For cell culture experiments, all inhibitors used were formulated in 100% DMSO. The MEKi, PD0325901, was obtained from Pfizer; fulvestrant was purchased from Sigma for cell-based experiments and pharmacy-grade drug was used for animal experiments; the protein Kinase B (AKT) inhibitor, MK-2206 was purchased from Chemietek; the pan-erbB inhibitor lapatinib was obtained from the developmental therapeutics program, NCI, and the Ribosomal S6 Kinase (RSK) inhibitor BI-D1780 was purchased from Enzo Life Sciences.

### Quantitative RT-PCR

Total RNA was extracted from cells (RNeasy, Qiagen) and cDNA synthesized (SuperScript® VILO, Life Technologies) and used for quantitative RT-PCR to determine the expression of genes of interest. Sequences for all primers utilized were obtained from primerbank (http://pga.mgh.harvard.edu/primerbank/), except for the ESR1 gene (accession NM_000125.3), for which primers were designed according to the sequence 5′–CCTGGGACTGCACTTGCT –3′and reverse 5′-CACAGCCCGAGGTTAGAGG-3′. Target gene expression was normalized to cyclophilin b, and data presented as fold-change relative to vehicle-only control.

### Cell proliferation Assays and Multiple Drug Effect Analysis

Doubling times were determined by counting cells at various stages of confluence using a coulter counter, and calculating doubling time (h) according to the formula, t*ln(2)/ln(A/Ao), where A is the cell number at time t; Ao is the initial cell number. The effect of drugs on cell proliferation was determined using the Sulforhodamine B (SRB) assay [24], and IC_50_’s determined by seeding cells at 1.5–4.5×10^4^ cells per ml into 96-well plates and approximately 8–16 h after, adding serial dilutions of drugs and incubating for three cell doublings (72–144 h) without replenishing media or drug. For combination studies, cells were treated with fulvestrant at a fixed dose of 200 nM, dilutions of MEKi, and the combination of both (using a fixed dose of fulvestrant combined with dilutions of MEKi at a range of doses encompassing the IC_50_). The nature of the drug interaction was evaluated using the Bliss additivity model [25], E_C_ = (E_A_+E_B)_ – (E_A_×E_B_). E_A_ and E_B_ are fractional inhibition of growth (relative to vehicle-only control) for drug A (fulvestrant), and drug B (MEKi) at specific concentrations. E_C_ is the expected fractional inhibition that predicts an additive interaction between A and B (combined fulvestrant and MEKi). If the experimentally measured fractional inhibition is greater than E_C_, the combination is greater than additive (synergistic). If the experimentally measured fractional inhibition is less than Ec, the combination is predicted to be antagonistic. Bliss additivity is the preferred method for analyzing drug interactions when one (or more) components are cytostatic (do not have a dose-response).

### Immunoblotting and Immunoprecipitation

Cells were treated with the indicated concentrations of drugs for the periods defined, and cell extracts obtained by solubilization in Tris-SDS denaturing lysis buffer. Extracts were resolved on SDS-PAGE gels, transferred to nitrocellulose and probed with relevant antibodies. For immunoprecipitation studies, drug treated SKOV3 cells were lysed in RIPA buffer, and 1 mg protein immunoprecipitated overnight with 4 µg of ERα antibody, followed by 2 hours incubation with 40λ protein A/G plus-agarose beads. Immunoprecipitated complexes were released from beads by boiling in Laemmli sample buffer and resolved on 7.5% acrylamide gels. Proteins immobilized onto nitrocellulose were immunoblotted for total ERα, ERα^118^ and ERα^167^.

### Cell Cycle Distribution by Flow Cytometry

To simulate estrogen deprivation conditions, SKOV3 cells were grown in phenol-free RPMI containing 10% charcoal stripped fetal bovine serum and treated with 1 µM MEKi for 24 hours. Adherent and nonadherent cells were harvested, fixed in 70% ethanol, permeabilized with 0.1% Triton X-100 and stained with 10 µg/ml propidium iodide (Sigma) in a PBS solution containing 1 µg/ml RNase A. Cell cycle acquisition and analysis was performed using the Becton Dickinson FACScan and Flowjo software, gating to remove debris and cellular aggregates.

### In vivo Validation

All mice used in this study were maintained in the Einstein Animal Facility. This Institutional Animal Welfare Assurance (A3312-01) is fully accredited by the Association for the Assessment and Accreditation of Laboratory Animal Care (AAALAC), February 22, 1983. All animals received humane care as per the Animal Welfare Act and the NIH “Guide for the Care and Use of Laboratory Animals”. Experimental protocols were reviewed and approved by the Einstein Institutional Animal Care and Use Committee, (Protocol No. 20100612), and all studies were performed according to the methods approved in this protocol.

The human ovarian cancer xenograft model, SKOV3 was established in nude mice as described previously [26], using early passage cells (approximately 3–5). Female nude mice were injected subcutaneously with 5×10^6^ cells per animal. MEKi was formulated as described previously [22] and administered daily by oral gavage at 5 mg/kg, except weekends. Clinical grade fulvestrant was administered subcutaneously at 5 mg/mouse twice weekly. Mice bearing tumors of approximately 150 mm^3^ (*n* = 5 per treatment group) were treated with either MEKi or fulvestrant alone, or the combination, whereby drugs were given concurrently (MEKi daily and fulvestrant on days 1 and 4). Control mice received vehicle alone. Mice were weighed twice weekly, and tumor dimensions also measured twice per week, and used to calculate tumor volume that was expressed relative to initial tumor volume. Mice were orally gavaged with saline to relieve transient hyperkeratosis and diarrhea that occurred in the combination-treated group.

## Results

### MEK Inhibition Leads to ERα Overexpression in ERα-positive Estrogen-dependent Ovarian Carcinoma Cells

**Figure 1:**
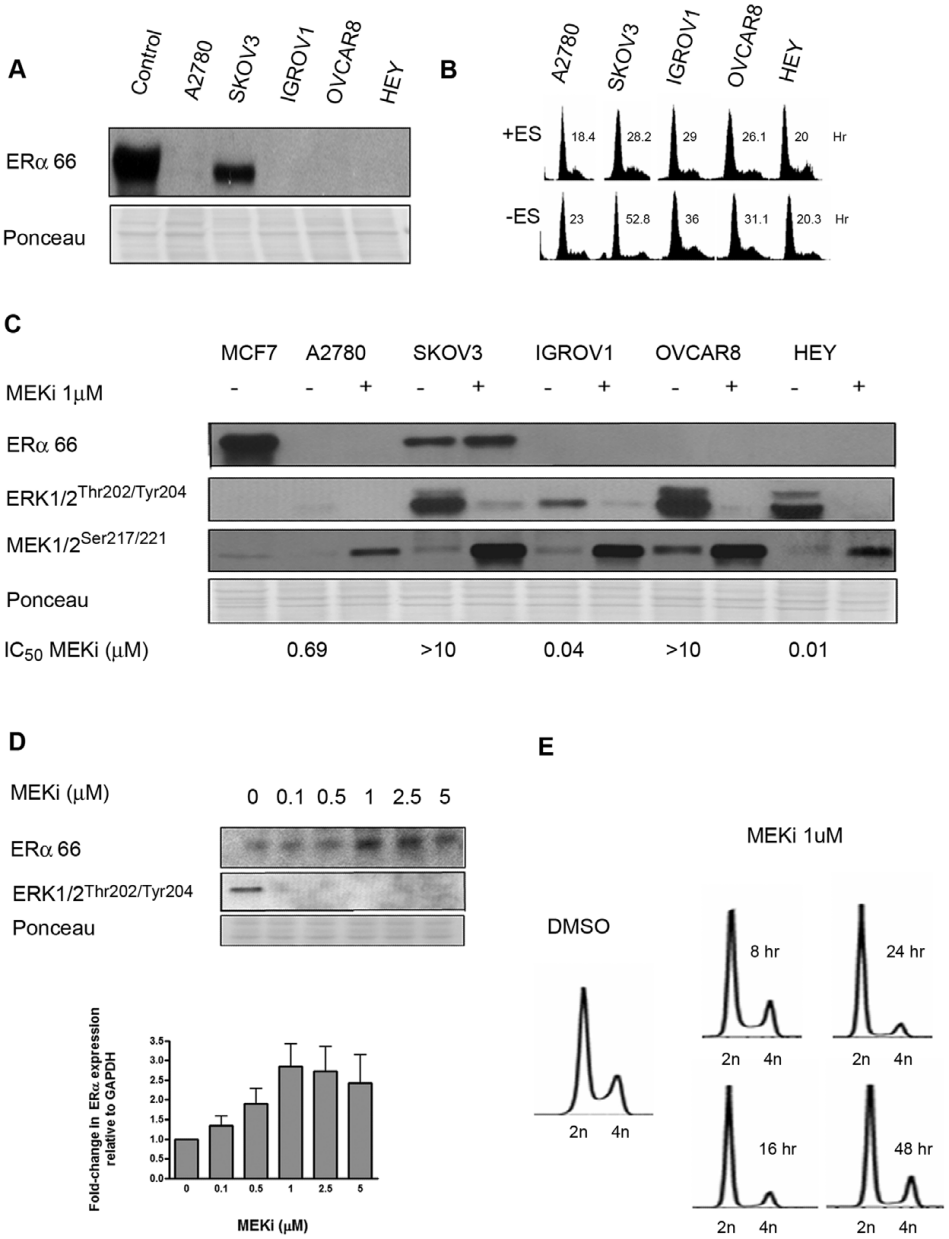
MEK Inhibition Increases ERα Expression in Human Ovarian Carcinoma Cells. (A) Expression of ERα protein in human ovarian cancer cell lines. MCF-7, a breast cancer cell line was used as positive control. All cell lines were treated with MEKi at 1 uM for 24 h. (B) The effect of estrogen deprivation on cell cycle. Cells were grown in phenol red-free charcoal stripped RPMI for 48 h to simulate ES-free conditions, and subsequently analyzed for cell cycle distribution and doubling time, as described in Materials and Methods. (C) The effect of MEK inhibition for 24 h on ERα expression and MAPK pathway activation in ovarian cancer cells. DMSO was used as the vehicle-only control. (D) Dose-dependent increase in ERα expression in SKOV3 cells by MEKi (24 h); and densitometric quantification relative to GAPDH. (E) Flow cytometric analysis of cell-cycle distribution at various time points indicates G1 arrest 24 h post MEKi treatment in SKOV3 cells.

In a panel ovarian cancer cell lines, SKOV3 was the only ERα expressing cell line among the five tested (Fig. 1A). Treatment of cell lines with 5-azacytidine, the demethylating agent, did not alter expression of ERα (data not shown), indicating that the lack of ERα expression in the majority of cell lines analyzed is not epigenetically controlled.

To determine the dependence of cellular proliferation on estrogen, we cultured the ovarian cell lines in phenol red-free, charcoal-stripped media; and quantified this effect on cell cycle distribution and cell proliferation (Fig. 1B). Estrogen withdrawal had the most impact in the ERα-positive cell line, SKOV3, as indicated by a 2-fold increase in doubling time and the prominence of G1 arrest (Fig. 1B & Fig. S1). These effects were almost entirely reversible with titration of ES back into ES-free media, indicating that was ES dependency, not the serum-depleted media that was responsible for this phenomenon. Growth suppression was not observed in the ER-negative cell line, A2780 (Fig. S1). Thus SKOV3 is an ERα expressing ovarian cancer cell line that is also estrogen-dependent for proliferation.

Since MAPK is known to regulate nongenomic ERα signaling [12], [13], we evaluated the effect of MEKi on ERα expression in our ovarian cancer cell panel. Two classes of response were observed: resistant (SKOV3, OVCAR8), and sensitive (A2780, Hey, and IGROV1). The known genotype, histology, and IC_50_ of the cell lines to MEKi are summarized in Table S1. Both SKOV3 and A2780 have hyperactive PI3K/AKT signaling due to mutations in PI3KCA and PTEN, respectively; however, their differential response to MEKi suggests that constitutively active AKT downstream is not prognostic of response to MEKi in ovarian cancer cells. Hey cells are *B-Raf* mutant and are hypersensitive to MEKi, consistent with previously published studies [22], [23].

Upon treatment with MEKi, target inhibition (dephosphorylation of ERK^T202/Y204^) and *Ras-*mediated feedback (apparent as increased phosphorylation of MEK^S271/221^_)_ was observed in all cell lines, even those with high intrinsic MAPK activity (Fig 1C) [27]. Thus, neither dephosphorylation of ERK, or phosphorylation of MEK are predictive of response to MEK inhibitors. MEKi caused a dose-dependent increase in ERα expression (approximately 2–3 fold increase at 24 h at 1 µM dose by densitometry analysis utilizing GAPDH normalization) that coincided with a significant G1 arrest at the same time point, as determined by flow cytometry (Fig. 1D–E). Changes in ERα expression by MEKi prior to 24 h were insignificant (data not shown). To substantiate the applicability of this model in other gynecologic malignancies, we also evaluated changes in ERα expression in the ERα-positive endometrial carcinoma cell line, Ishikiawa (Fig. S2). Consistent with our observations in ovarian cancer cells, MEKi treatment was associated with estrogen receptor overexpression.

### ERα Overexpression is Associated with Increased Phosphorylation at known MAPK-Regulatory Sites, and Transcription of known ER-responsive Genes

**Figure 2:**
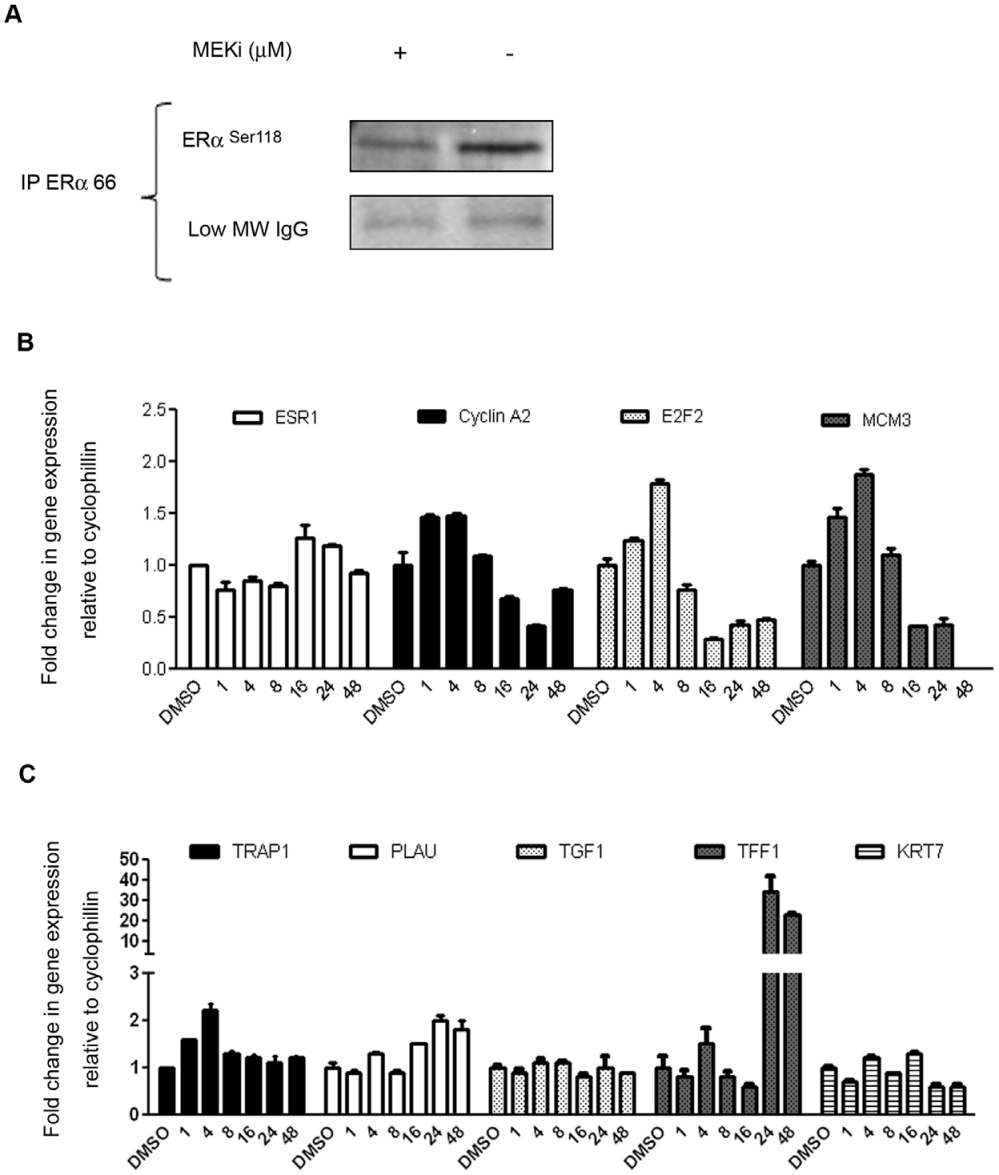
ERα overexpression is associated with MAPK-dependent phosphorylation, cell-cycle arrest and transactivation of ER-regulated genes. (A) MEKi treatment for 24 h increases ERα phosphorylation at Serine 118 in ER-immunoprecipitated SKOV3 lysates. (B) The effect of MEKi on *ESR1* and cell cycle regulatory gene expression, depicting upregulation and suppression, respectively. (C) The effect of MEKi on expression of selected ER-regulated genes in SKOV3 cells. Treatment with MEKi was for 24 h, and mRNA expression was carried out by qRT-PCR as described in Materials and Methods.

To determine whether the changes in ERα expression were correlated with altered phosphorylation at known MAPK-regulatory sites (ERα^S118^ ^and^ ^S167^), we immuno-precipitated total ERα from MEKi-treated SKOV3 cells. ERα^S118^ is a known phosphorylation site for activation by ES, which subsequently promotes nuclear localization of ERα, and recruitment of coactivators and promotors for transactivation of target genes. ERα^S118^ can also be activated in a ligand (ES)-independent manner by MAPK, GSK-3, IKKα, CDK7, and mTOR/p70S6K [28], [29]. After MEKi treatment for 24 hours, a 1.5-fold increase in ERα^S118^ was observed relative to both total ERα and IgG (Fig. 2A). ERα^S167^, another phosphorylation site that is regulated by RSK, as well as AKT, mTOR and S6K [8] [30], was unchanged upon drug treatment (not shown). Since MEKi is a highly specific, non-ATP competitive signaling inhibitor, it is likely most that the increased phosphorylation of ERα^S118^ and the lack of changes in ERα^S167^ observed after treatment is due to direct suppression of MAPK - ERK signaling.

To investigate the effect of increased ERα phosphorylation by MEKi on genomic ER-signaling, we determined the expression of ES-regulated cell cycle genes and genes known to affect cellular differentiation and migration: specifically, TRAP1, PLAU, TGF1, TFF1, KRT7 [31], [32]. MEKi modestly increased (1.5-fold) transcription of the ER gene, *ESR1,* by 16 h in SKOV3 cells (Fig. 2B). This was associated with a decrease in cell cycle regulatory genes after 16 h, consistent with the G1 arrest shown in Fig. 1E. Thus, modulation of ER gene, protein expression, and phosphorylation status correlate with proliferative arrest. There were modest increases in the expression of *plau*, an ER-regulated gene involved in extracellular matrix remodeling, and a modest decrease in *KRT7* an ER-regulated keratin whose function is involved in DNA synthesis. These changes occurred primarily at 24–48 h post-dosing, consistent with the time point at which increased expression of ERα by MEKi was noted. Of interest was the dramatic up-regulation of another ER-regulated gene, *TFF1* [33], trefoil factor 1 (Fig. 2C), which is normally expressed in the epithelium of the breast and ovary [34], [35]. It is also expressed in gastric mucosal cells, where its function is to stabilize the mucosal layer and protect tissue from cellular injury [36]. The role of *TFF1* in tumorigenesis is controversial, but it is a marker of cellular differentiation, and in some contexts has tumor suppressive activity [37]. Thus, transactivation of *TFF1* by MEKi is consistent with our observed activation of ERα and may denote a favorable change in differentiation status.

### MEKi Mediated ERα Overexpression is Independent of AKT

**Figure 3:**
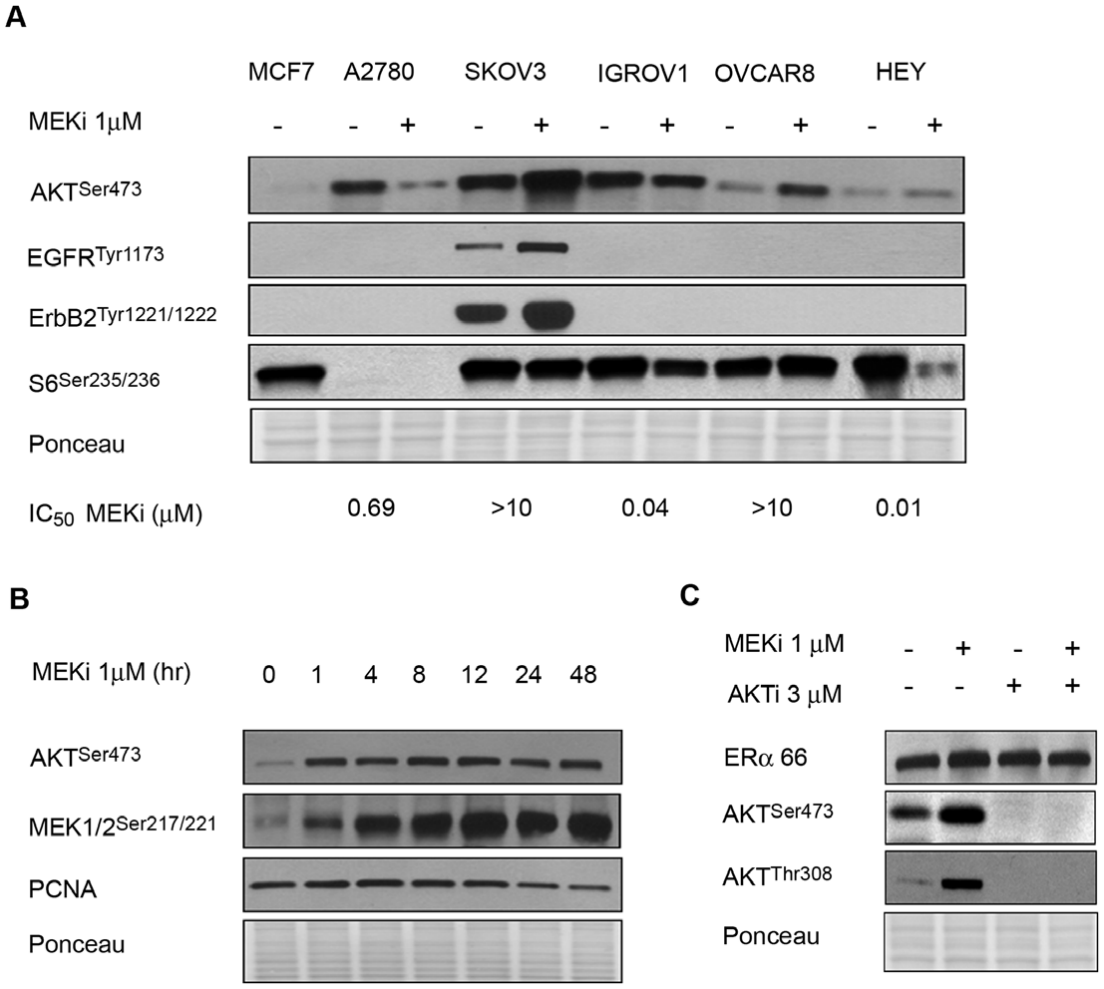
MEKi-mediated overexpression of ERα is AKT independent. (A) MEKi-mediated changes in AKT phosphorylation, and not basal phosphorylation, are prognostic of drug sensitivity. Increased phosphorylation of erbB-family receptors in SKOV3 cells also correlate with resistance to MEKi, while S6 dephosphorylation predicts sensitivity to MEKi. Mean IC_50_’s for MEKi are shown. (B) Temporal dissociation of pAKT and pMEK with ERα overexpression after treatment with 1 µM MEKi. (C) AKT inhibition combined with MEKi does not reverse ERα overexpression in SKOV3 cells. Cells were treated with the specified inhibitors for 24 h.

The effect of MEKi on AKT signaling in ovarian cancer cells was evaluated. The basal level of AKT phosphorylation was not predictive of response (Fig. 3A). AKT phosphorylation by MEKi is prognostic of response in human lung carcinoma [27], and in the ovarian cancer cell lines shown in Fig. 3A, increased AKT^S473^ after MEKi correlated with resistance in SKOV3 and OVCAR8. This increase in AKT activity after MEKi may be a feedback effect via erbB family members, including EGFR, and Her 2/neu, as previously reported [38], [39]. Thus, the basal expression of erbB proteins and their activation by MEKi may mediate increased AKT activity and possibly contribute to resistance, as seen in the case of SKOV3.

We investigated the temporal phosphorylation of AKT and its association with ERα expression from 0–24 h after MEKi. As shown in Fig. 3B, MEKi caused rapid feedback onto the MEK/Ras pathway, as demonstrated by increased MEK^S217/221^ phosphorylation within 1 h that plateaued by 4 h. Increased phosphorylation of AKT^S473^ was also observed within 1 hr [27]. The temporal dissociation between AKT activation (noted at one hour) and changes in ERα expression by MEKi (at 24 hours) suggest that the two events are mechanistically dissociated. To substantiate this further, we treated SKOV3 cells with MEKi in the presence of the allosteric AKT inhibitor MK-2206 (AKTi) [40]. As shown in Fig. 3C, AKTi inhibited phosphorylation at T308 and S473, as well as its downstream effector, 4EBP1 (data not shown); however ERα expression was unaffected when AKT was suppressed, either by AKTi alone, or in combination with MEKi. Therefore, MEKi-mediated overexpression of ERα is AKT-independent.

Phosphorylation of ribosomal protein, S6^S235/236,^ a component of the 40S ribosomal subunit that is downstream of mTORC1, occurs in response to mitogenic stimulation of MAPK, via RSK [41]. Our studies in lung cancer have shown that drug-induced changes at S6^S235/236^ predict response to MEKi [22], [27]. This also holds true for ovarian cancer cell lines, since IGROV1 and HEY that are hypersensitive to MEKi, have decreased phosphorylation of S6 upon drug treatment (Fig. 3A).

Suppression of PCNA, (a marker of proliferation) by 24 h after MEKi-treatment, coincided with the onset of ERα overexpression, suggesting a relationship between proliferative arrest and altered expression and activity of ERα.

### MEKi-mediated ERα Overexpression is Independent of erbB and is ERK-dependent

**Figure 4:**
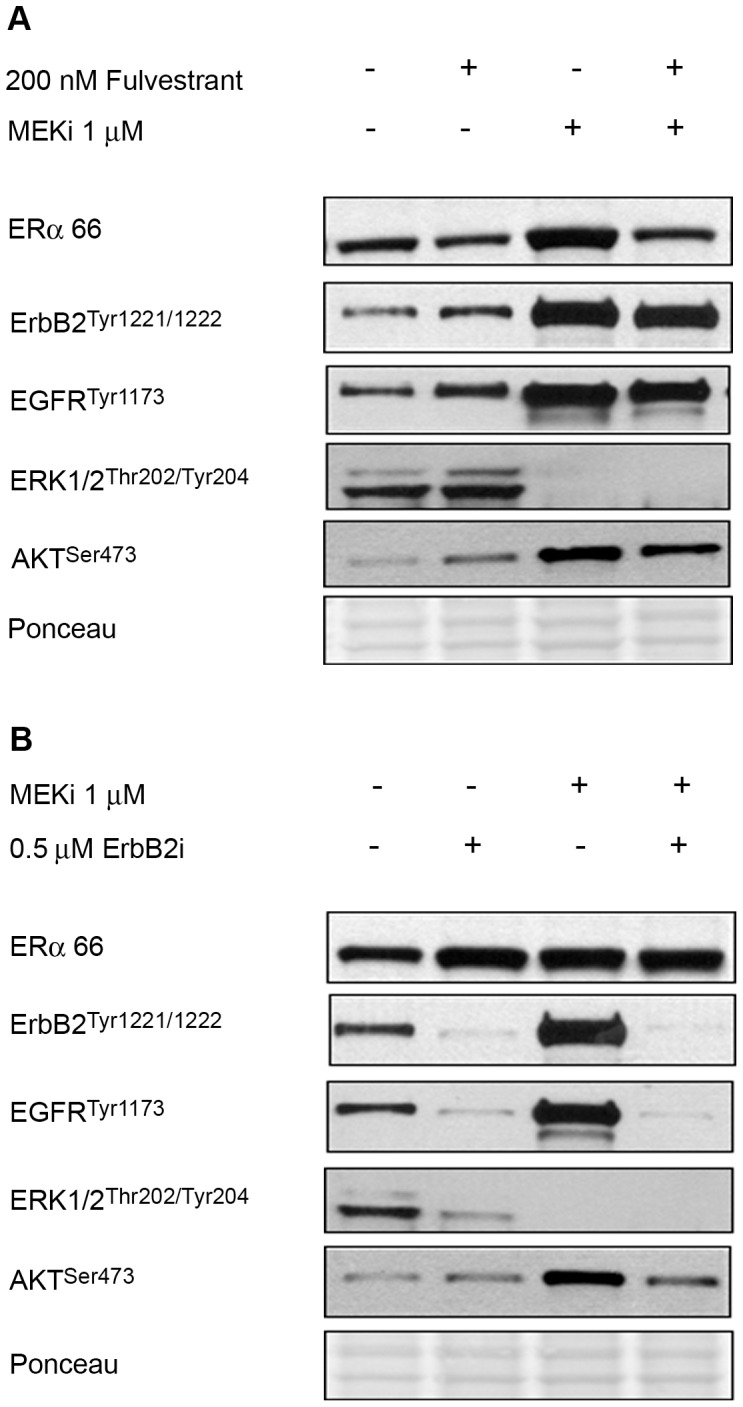
MEKi-mediated ERα overexpression is independent of erbB activity but MAPK-dependent. (A) The ERα antagonist fulvestrant prevents MEKi-mediated ERα overexpression, and partially suppresses the phosphorylation of erbB2 and EGFR by MEKi. (B) Overexpression of ERα by MEKi is erbB-independent, since the pan-erbB inhibitor lapatinib (erbBi) does not prevent ERα overexpression by MEKi treatment. Cells were treated with inhibitors for 24 h.

We next determined the effect of ERα receptor inhibition (using the antagonist fulvestrant) on erbB feedback by MEKi (Fig. 4A). Doses of drugs that result in potentiation, (as summarized in Table 1 and described in Methods), were utilized. As expected, fulvestrant alone suppressed ERα, and this was sustained in the presence of MEKi. In addition, the combination of fulvestrant and MEKi partially suppressed feedback activation of erbB2, EGFR and AKT that was observed with single agent MEKi. Therefore, receptor tyrosine kinase (RTK) activation, such as erbB family members, may contribute to ERα potentiation; and may be mechanistically involved in mediating the synergy observed between these two drugs (Table 1 & Fig. S3).

**Table 1:** Potentiation of MEKi efficacy by the estrogen receptor antagonist Fulvestrant in ERα-positive cancer cell lines.

CELL LINE	ERα Status	Effect of Fulvestrant (200 nM) on Growth¶	Effect of MEKi (10–0.3 µM) on Growth	Effect of Combination (Expected additive) effect)^§^	Effect of Combination(Observed)
**SKOV3**	+	115% Increased proliferation)	30–10% inhibition	**18–5%**	**32–25%**
**A2780**	−	20% inhibition	80–30% inhibition	83–48%	80–31%
**Ishikawa**	+	1% Inhibition	40–10% Inhibition	**40–28%**	**47–32%**

The predicted additive effect was determined by applying the Bliss additivity model**^§^** [25]. A greater than additive or synergistic interaction (observed effect exceeds the expected effect) was noted only in the ERα- expressing cell lines, SKOV3 and Ishikawa. Conversely, A2780, ERα-negative ovarian carcinoma cells exhibited antagonism between MEKi and fulvestrant.

¶ As determined by SRB assay.

SKOV3 cells were treated with the pan-erbB inhibitor lapatinib to further explore the potential role of erbB/EGFR in mediating changes in ERα after MEKi treatment. Since lapatinib acts upstream of MAPK and PI3K, and has the potential to suppress both pathways, it may also increase ERα expression in the same manner as MEKi. As shown in Fig. 4B, lapatinib increased ERα expression to the same degree as MEKi, and strongly suppressed erbB2, EGFR and ERK phosphorylation. Although there was a minor effect on AKT, the data presented in Fig. 3C clearly do not support a role for AKT as the mediator of ERα modulation by MEKi. Thus, suppression of MAPK – ERK is likely to cause the changes in ERα expression and activity after MEKi treatment.

Similar to ER, activated ERK can participate in cytoplasmic, non-genomic signaling via activation of RSK. Since RSK has been shown to directly regulate ER via phosphorylation on S167, we probed the involvement of the cytoplasmic ERK-RSK pathway in mediating ERα overexpression in response to MEKi, using the specific inhibitor RSK inhibitor, BI-D1780 (RSKi). RSKi specificity is evident from the suppression of its downstream effectors, AKT and GSK3, as described [29]. RSKi alone suppressed total ERα and its phosphorylation (Fig. S4), in contrast to what we observe with MEKi alone. This suggests that the MEKi-mediated effects on ERα are RSK-independent. Therefore, this is further evidence implicating ERK as the kinase likely to mediate changes in ERα expression and phosphorylation by MEKi in SKOV3 cells. These data, coupled with Fig. 3C, provide evidence that ERα overexpression by MEKi is independent of ErbB2, EGFR, AKT and RSK, and is mediated via ERK suppression.

### Combined Fulvestrant and MEKi are Synergistic in ERα Expressing Cancer Cell Lines

To exploit the phenomena of ERα overexpression, we evaluated the efficacy of MEKi in combination with the ER receptor antagonist, fulvestrant, in the ERα-positive cancer cell lines (SKOV3 and Ishikawa). A2780, an ERα-negative ovarian carcinoma cell line, was used as a negative control. Fulvestrant alone mildly increased the proliferation of SKOV3 cells; however, the combination of fulvestrant and MEKi negated this, and potentiated sensitivity to MEKi (Table 1, Fig. S3). This resulted in a greater than additive (synergistic) drug interaction, as predicted by Bliss additivity [25]. In the ERα-negative cell line, A2780, the same combination was antagonistic; therefore, this drug combination may have utility in ERα-expressing malignancies. To further validate our hypothesis that MEKi-mediated potentiation of ERα is important in mediating the synergy and anti-tumor effect of combined MEKi and fulvestrant, we evaluated the expression of ER-responsive genes after treatment with MEKi or fulvestrant, or the combination of both (Fig. 2B & C). As expected, the drugs had different effects on different genes; however an interaction between MEKi and fulvestrant was noted for TFF1, an estrogen-responsive gene [42]. MEKi significantly increased its expression (consistent with the increased expression and phosphorylation of ERα-regulated genes); however this increase was prevented by fulvestrant (Fig. S5). Though we do not know if this change is causative, or simply an association with the observed synergy, it is an interesting observation that highlights the ability of fulvestrant to prevent modulation of TFF1 by MEKi.

### Fulvestrant Potentiates the Activity of MEKi in an ERα-expressing Ovarian Tumor Xenograft Model

**Figure 5:**
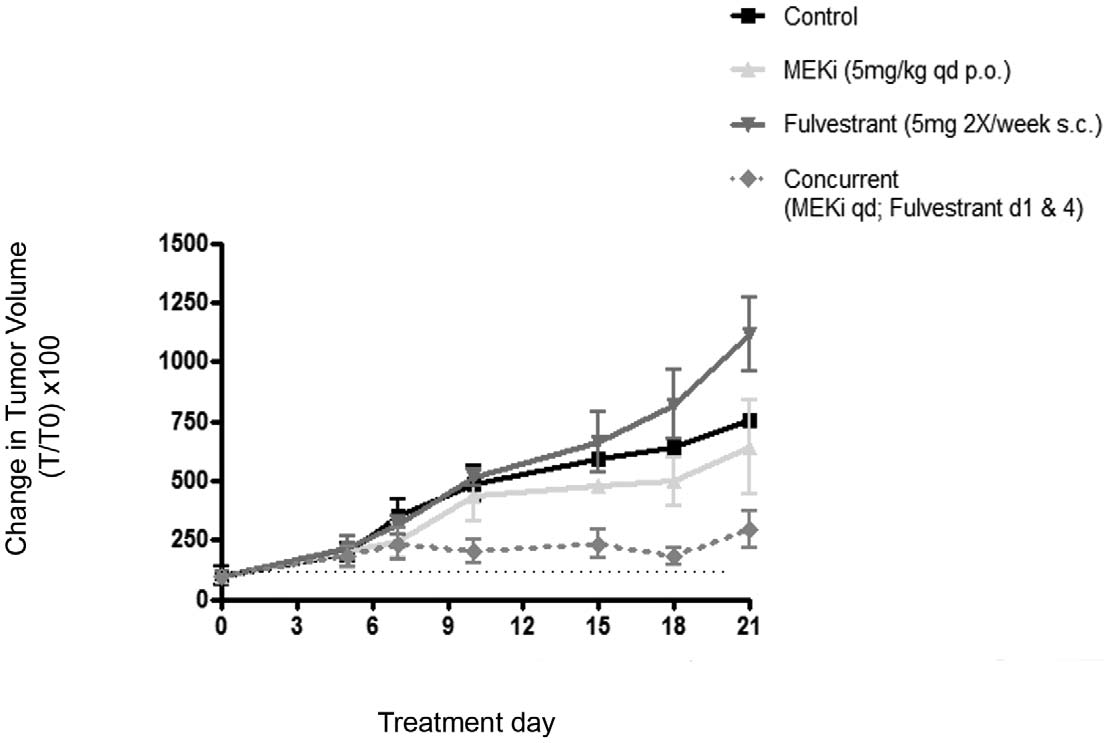
The concurrent combination of MEKi and fulvestrant suppresses SKOV3 tumor xenograft growth. Single agent fulvestrant weakly stimulated tumor growth relative to vehicle, and MEKi had weak anti-tumor activity; however the concurrent combination of fulvestrant and MEKi induced tumor regressions that were statistically significantly different from either MEKi alone (*P = 0.02, unpaired t-test), or fulvestrant (**P = 0.002, unpaired t-test) at day 18. After three weeks, animals in the treatment groups other than combination were euthanized due to tumor burden. Asterisks denote the level of significance. Data are expressed as percent change in initial tumor volume (T_0_). The dashed horizontal black line represents initial tumor volume.

The combination of fulvestrant and MEKi was evaluated in nude mice bearing SKOV3 xenografts (Fig. 5). The mean tumor volume at dosing initiation was approximately 150 mm^3^. Similar to what we observed in cell culture, fulvestrant alone increased tumor growth, while MEKi alone had weak activity; however the combination of both potently suppressed tumor growth. This effect was sustained for three weeks post dosing, when animals in other groups had to be euthanized due to tumor burden. The mean tumor volume of the combination treatment was statistically significantly smaller than either single agent alone by 18 days post-treatment (*p* = 0.02, MEKi versus combination; *p* = 0.002, fulvestrant versus combination). Moreover, the combination regimen was well tolerated. Noted toxicities including hyperkeratosis and diarrhea were resolved with topical antibiotic application and oral hydration, respectively. Thus, these data strongly support a role for combined MEKi and fulvestrant therapy as a promising regimen with impressive anti-tumor efficacy in an ERα-positive EOC tumor model.

## Discussion

In this proof of concept study, we have shown that the combination of MEKi and fulvestrant has synergistic activity *in vitro*, and has promising anti-tumor efficacy *in vivo,* in ERα positive ovarian cancer. Furthermore, we demonstrate that MEKi-mediated overexpression of ERα is due to ERK suppression. The precise mechanism by which this occurs is likely to be multifactorial; however it does not appear to be mediated by feedback signaling that activates RTK, and AKT. Thus, the ability of MEKi (and other drugs upstream of ERK, such as lapatinib) to increase the expression of ERα can be therapeutically exploited to render cancer cells sensitive to endocrine therapy, irrespective of their response to MEKi alone.

Although fulvestrant is largely perceived to be an ER-antagonist, it has recently emerged that at high doses it can act as a partial agonist in some cell types, and promotes shuttling of the ER to the plasma membrane, resulting in complex formation with the IGF-1-receptor (IGF-1-R) [43]. This prevents degradation of the ER and facilitates its participation in non-genomic signaling, including MAPK activation. It has also been demonstrated in MCF-7 cells that long-term tamoxifen treatment enables ER translocation from the nucleus to the plasma membrane and subsequent activation of EGFR and IGFR pathways [44]. Although we have not evaluated these phenomena as mechanism for MEKi-induced ERα overexpression herein, it is a plausible explanation. This potential agonist activity of fulvestrant may also provide a mechanistic rationale for the synergy between fulvestrant and MEKi, whereby an ER - IGFR complex at the plasma membrane could confer increased cellular dependency on MAPK that would render cells more sensitive to MEKi. This hypothesis is currently being evaluated.

There is increasing appreciation for non-genomic signaling as a major driver of tumorigenesis, as discussed above in the case of ER-signaling. This model also applies to MAPK signaling as recently illustrated in the clinical evaluation of the B-Raf inhibitor, PLX4032, whereby clinical responses were associated with 80% dephosphorylation of cytoplasmic, but not nuclear ERK [45]. This supports the paradigm that cytoplasmic (non-genomic) signaling events involving MAPK, rather than transcriptional activity, confer potent tumorigenicity, at least in *Raf* mutant tumors.

In the clinical setting, selecting a targeted patient cohort that will derive maximum benefit from anti-tumor strategies is paramount in rational drug design. Type 1, or low grade ovarian carcinoma, arises from low malignant potential tumors, and harbor a higher frequency of *Ras* and *Raf* mutations (approximately 60%, with one study identifying 30% mutation rates in either gene respectively [4], [46]), as well as a high percent of ERα expression (approximately 60–80%) [47]. Although low grade ovarian cancer only accounts for 9% of all EOC [48], it is chemo-resistant relative to its high-grade counterpart [49]. Therefore, identification of novel agents that are more effective in this disease, and particularly in this subtype, is an active and worthy area of clinical investigation.

Although ERα positive malignancies are conventionally associated with improved prognosis and sensitivity to AET, exploitation of ERα as a treatment strategy has not gained wide acceptance in ovarian cancer care despite known expression of the receptor [9], [10]. In the metastatic or recurrent setting, ovarian cancer is considered hormonally resistant, based on early phase II trials showing poor response to tamoxifen in an unselected patient population [18]. Even when selecting for ERα-positive ovarian or endometrial cancer, fulvestrant treatment had a response rate of 8–16% [16], [50]. While these results may seem discouraging, one should consider that approximately one-third of ERα-positive breast cancers are resistant to AET, and 10% of ERα-negative breast cancer respond to tamoxifen [51]. In breast cancer cell lines, ERα-negative status may be induced by hyperactivation of RTK’s leading to MAPK activation [52] and epigenetic changes [53], [54]. Restoration of ERα expression in breast cancer using MEKi is associated with response to AET [52]. Therefore, there is documented interplay between MAPK and ER-signaling in tumorigenesis that may account for hormonal resistance, but also may be exploited for therapeutic development.

The ability of cancer cells to respond to and counteract the effects of therapeutics that threaten their survival is a well-documented phenomenon. Signaling pathways have evolved with innate adaptive abilities to form a regulatory ‘circuit’ via positive and negative feedback. This circuitry that is hard-wired in non-malignant cells is possibly amplified in cancer genomes that are genetically more ‘plastic’. Thus, feedback is an intrinsic response to signaling inhibitors that contributes to acquired resistance, or ‘adaptive resistance’. The challenge is to map the effects of different classes of signaling inhibitors on adaptive feedback pathways and ultimately exploit this information in the rational design of combination therapies, as demonstrated here.

## Supporting Information

Figure S1 **Estrogen-dependent proliferation occurs in ERα-expressing ovarian carcinoma cells.** Estrogen depletion, (by culturing cells in phenol-free media), suppressed the growth of ERα-positive SKOV3 cells by two-fold, but had minimal effect on ERα -negative A2780 cells. Titration of estradiol (E2) back into growth media reversed the phenomenon and was dose-independent. PF = Phenol free media containing charcoal-absorbed serum. RPMI = Regular media containing 10% fetal bovine serum.
(PPT)

Figure S2 **Over-expression of ERα by MEKi in the ERα positive endometrial carcinoma cell line, Ishikawa.** Cells were treated for 24 hours with MEKi.
(PPTX)

Figure S3 **Dose-response curves generated from SRB-based proliferation assays (see Materials and Methods) for single-agent MEKi, fulvestrant and the combination of both, demonstrating potentiation (greater than additive/synergistic cytotoxicity).** Cells were treated according to the experimental details described in Table 1.
(PPTX)

Figure S4 **MEKi-mediated effects on ERα are RSK-independent in SKOV3 cells.**
(PPTX)

Figure S5 **The Effect of fulvestrant and MEKi (alone and in combination) on ES-regulated gene expression in SKOV3 cells after treatment for 24 h.** Refer to materials and methods for experimental details.
(PPTX)

Table S1 **Sensitivity of Human Ovarian Carcinoma Cell Lines to MEKi.**
(PPT)
