# Correction: Exploiting MEK Inhibitor-Mediated Activation of ERα for Therapeutic Intervention in ER-Positive Ovarian Carcinoma

**DOI:** 10.1371/journal.pone.0151750

**Published:** 2016-03-11

**Authors:** June Y. Hou, Alicia Rodriguez-Gabin, Leleesha Samaraweera, Rachel Hazan, Gary L. Goldberg, Susan Band Horwitz, Hayley M. McDaid

The third author’s name is spelled incorrectly. The correct name is: Leleesha Samaraweera. The correction citation is: Hou JY, Rodriguez-Gabin A, Samaraweera L, Hazan R, Goldberg GL, Horwitz SB, et al. (2013) Exploiting MEK Inhibitor-Mediated Activation of ERα for Therapeutic Intervention in ER-Positive Ovarian Carcinoma. PLoS ONE 8(2): e54103. doi:10.1371/journal.pone.0054103.
