# Supplementary figures and images for: Exploiting MEK Inhibitor-Mediated Activation of ERα for Therapeutic Intervention in ER-Positive Ovarian Carcinoma
Source: PLoS One. 2013 Feb 4;8(2):e54103. doi: 10.1371/journal.pone.0054103 (PMC3563537; doi:10.1371/journal.pone.0054103)

## Slide 1
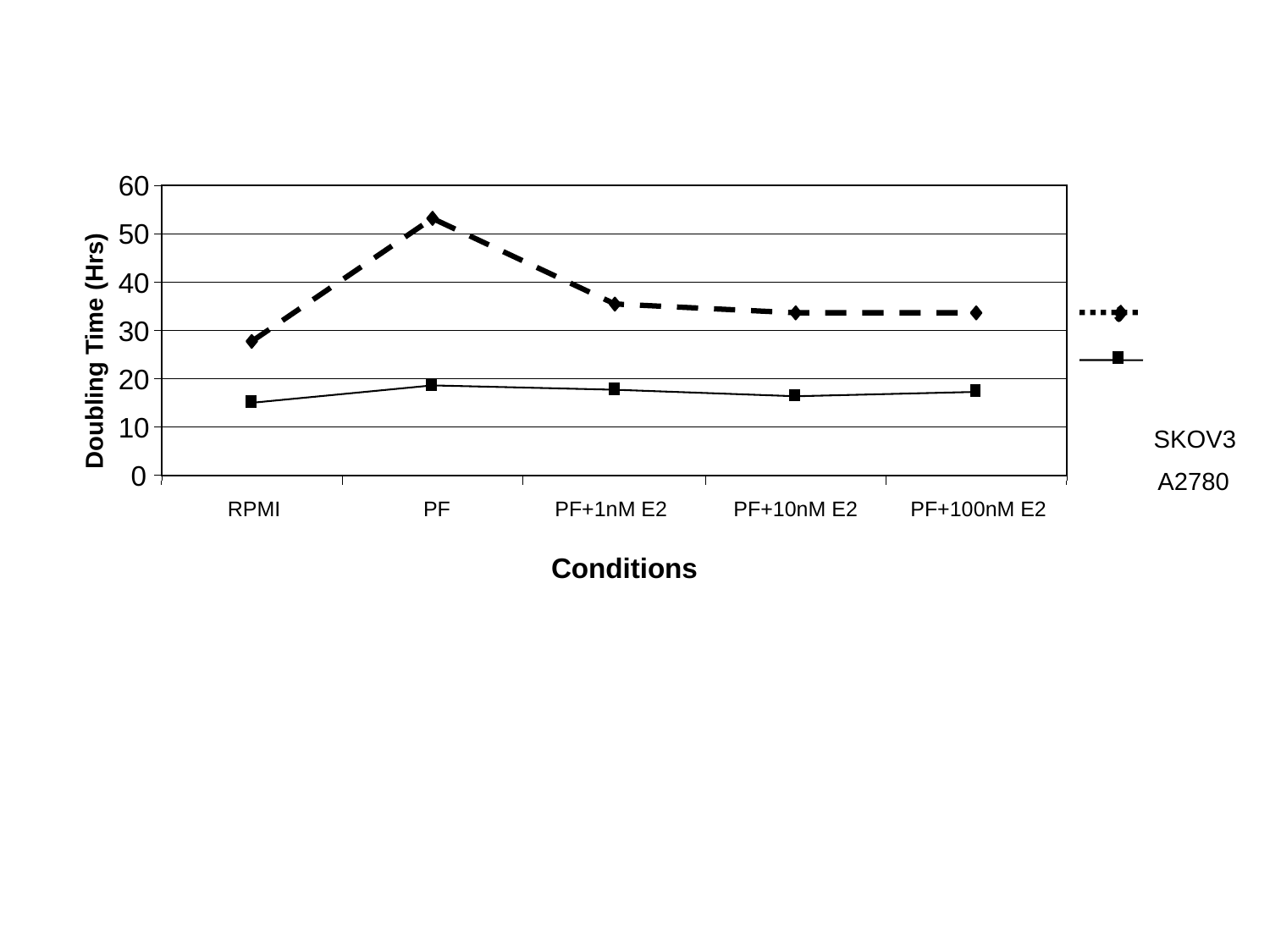

60
50
40
30
Doubling Time (Hrs)
20
10
SKOV3
0
A2780
RPMI
PF
PF+1nM E2
PF+10nM E2
PF+100nM E2
Conditions

Supplement: Figure S1 — Estrogen-dependent proliferation occurs in ERα-expressing ovarian carcinoma cells. Estrogen depletion, (by culturing cells in phenol-free media), suppressed the growth of ERα-positive SKOV3 cells by two-fold, but had minimal effect on ERα -negative A2780 cells. Titration of estradiol (E2) back into growth media reversed the phenomenon and was dose-independent. PF = Phenol free media containing charcoal-absorbed serum. RPMI = Regular media containing 10% fetal bovine serum. (PPT) [file pone.0054103.s001.ppt]

## Slide 1
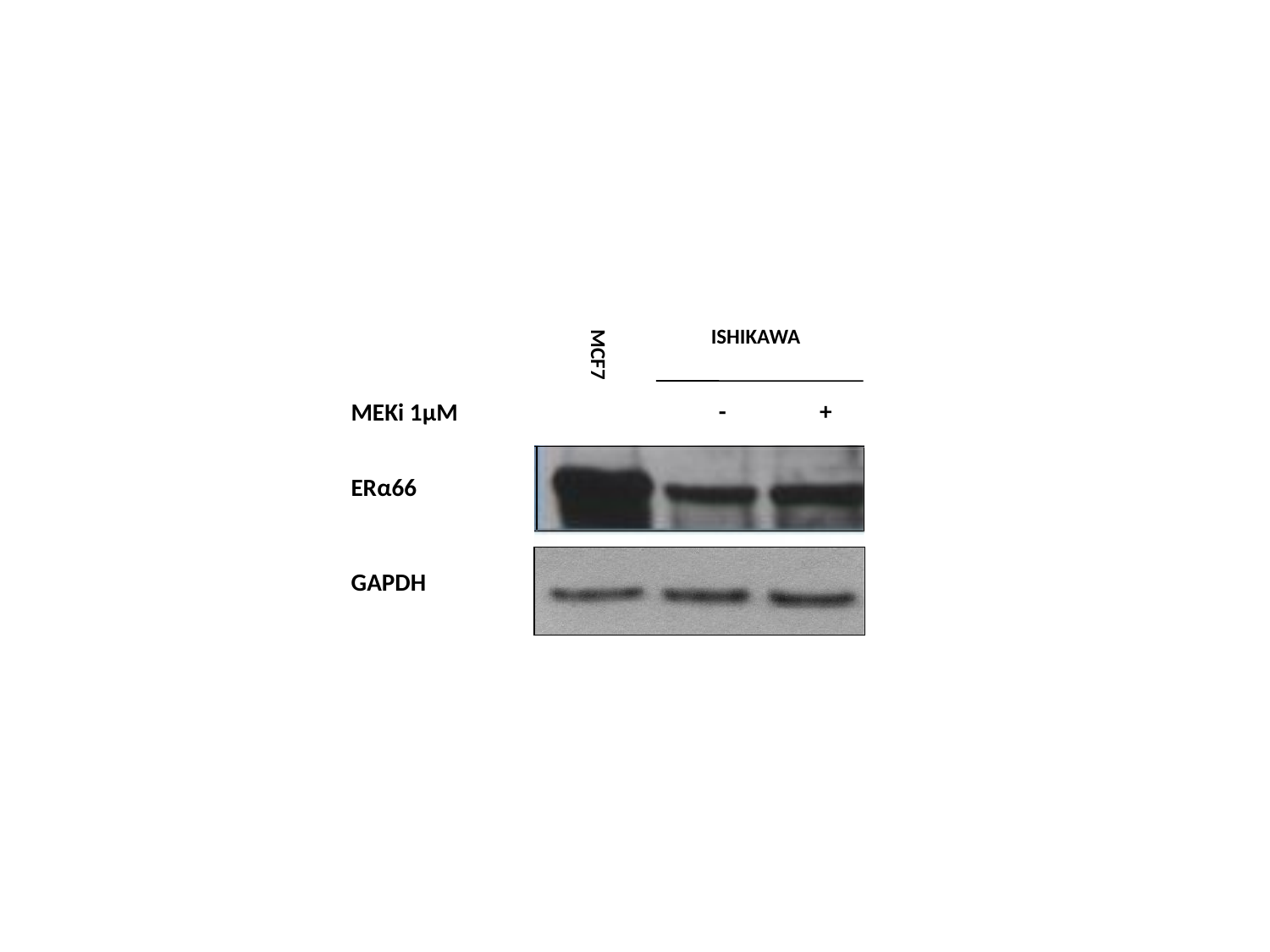

ISHIKAWA
MCF7
- +
MEKi 1μM
ERα66
GAPDH

Supplement: Figure S2 — Over-expression of ERα by MEKi in the ERα positive endometrial carcinoma cell line, Ishikawa. Cells were treated for 24 hours with MEKi. (PPTX) [file pone.0054103.s002.pptx]

## Slide 1
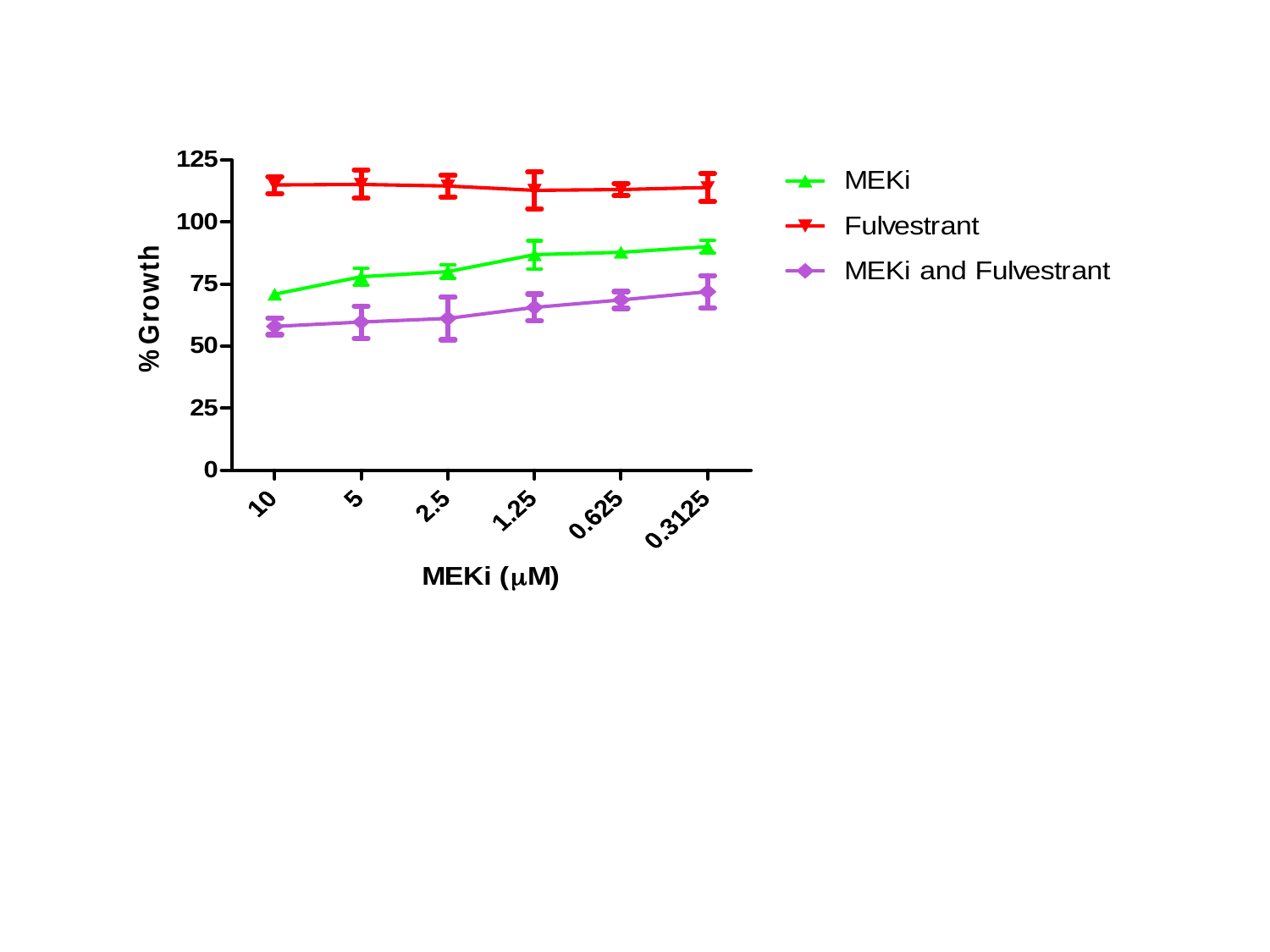

Supplement: Figure S3 — Dose-response curves generated from SRB-based proliferation assays (see Materials and Methods) for single-agent MEKi, fulvestrant and the combination of both, demonstrating potentiation (greater than additive/synergistic cytotoxicity). Cells were treated according to the experimental details described in Table 1. (PPTX) [file pone.0054103.s003.pptx]

## Slide 1
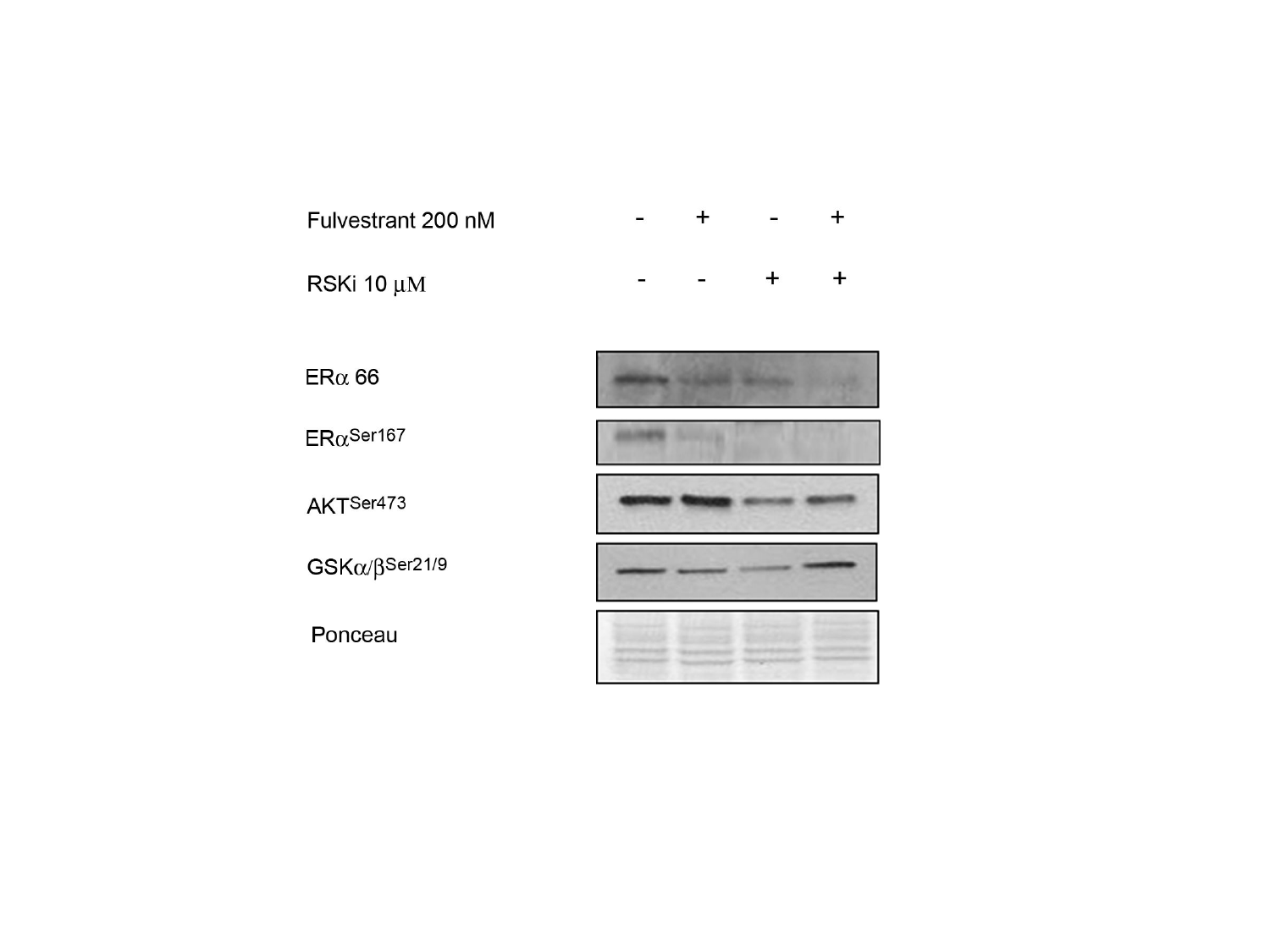

Supplement: Figure S4 — MEKi-mediated effects on ERα are RSK-independent in SKOV3 cells. (PPTX) [file pone.0054103.s004.pptx]

## Slide 1
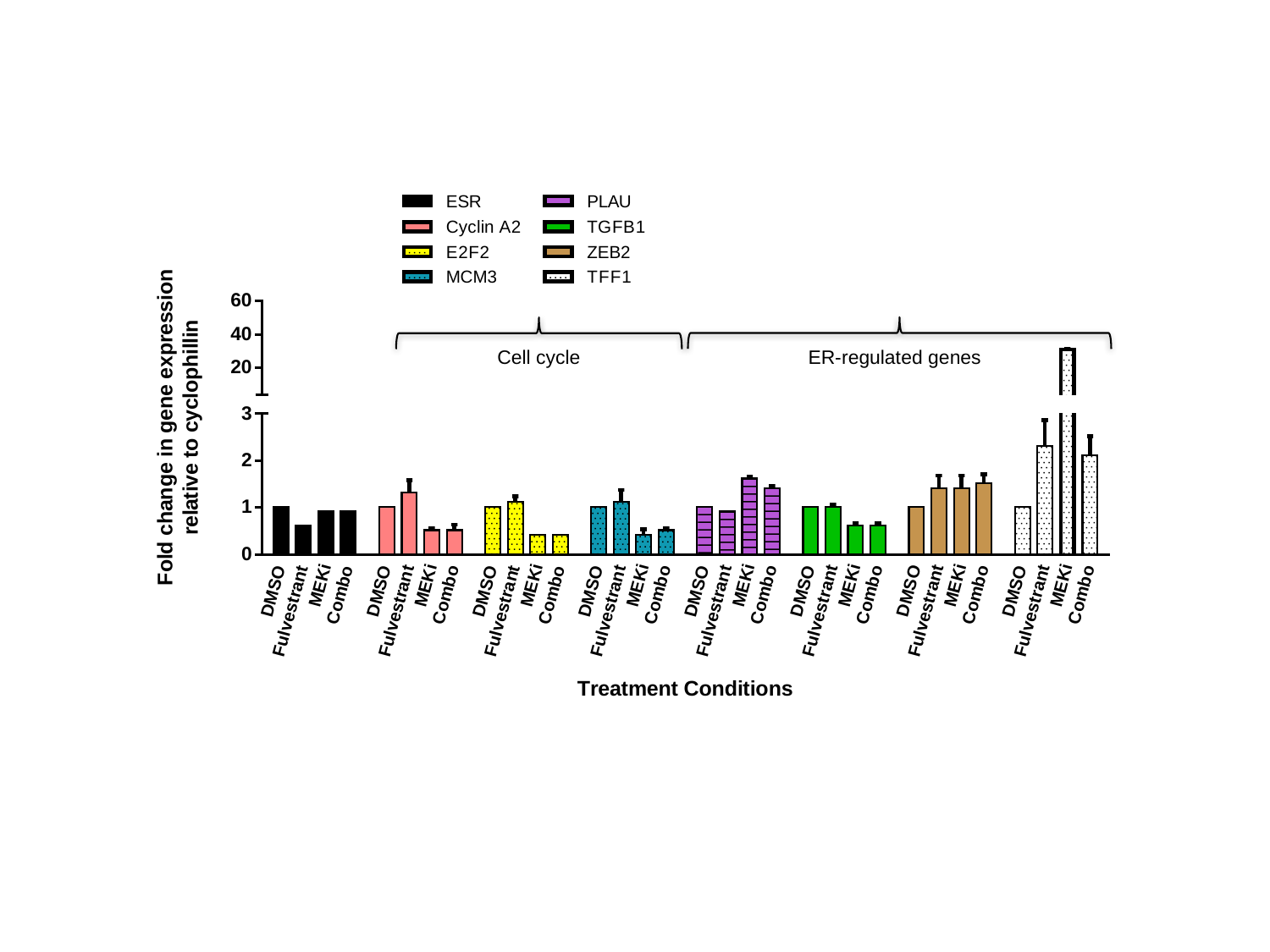

Cell cycle
ER-regulated genes

Supplement: Figure S5 — The Effect of fulvestrant and MEKi (alone and in combination) on ES-regulated gene expression in SKOV3 cells after treatment for 24 h. Refer to materials and methods for experimental details. (PPTX) [file pone.0054103.s005.pptx]
