# Supplementary material for: Exploiting MEK Inhibitor-Mediated Activation of ERα for Therapeutic Intervention in ER-Positive Ovarian Carcinoma
Source: PLoS One. 2013 Feb 4;8(2):e54103. doi: 10.1371/journal.pone.0054103 (PMC3563537; doi:10.1371/journal.pone.0054103)
Supplement: Table S1 — Sensitivity of Human Ovarian Carcinoma Cell Lines to MEKi. (PPT) [file pone.0054103.s006.ppt]

## Slide 1
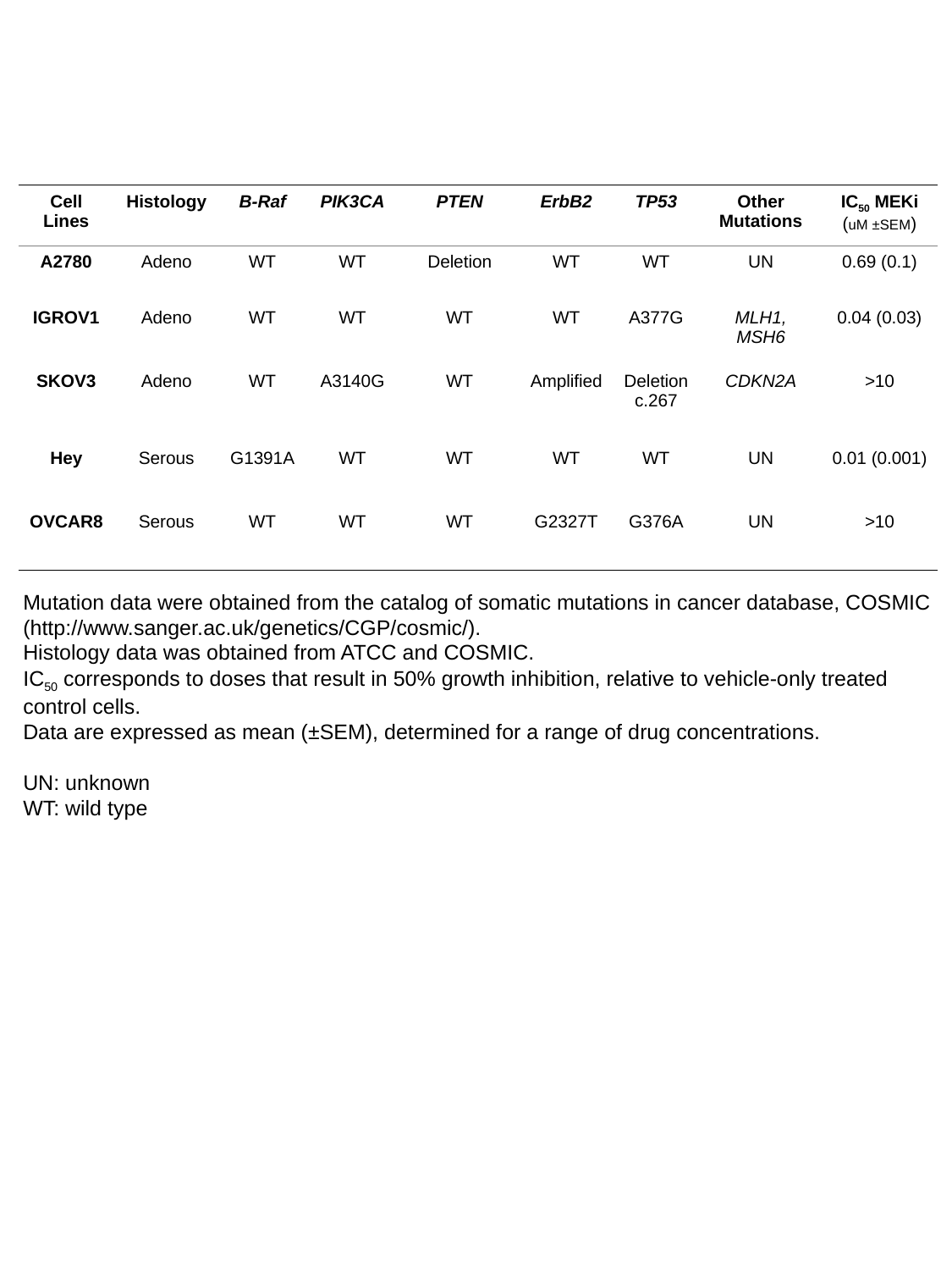

| Cell Lines | Histology | B-Raf | PIK3CA | PTEN | ErbB2 | TP53 | Other Mutations | IC50 MEKi (uM ±SEM) |
| --- | --- | --- | --- | --- | --- | --- | --- | --- |
| A2780 | Adeno | WT | WT | Deletion | WT | WT | UN | 0.69 (0.1) |
| IGROV1 | Adeno | WT | WT | WT | WT | A377G | MLH1, MSH6 | 0.04 (0.03) |
| SKOV3 | Adeno | WT | A3140G | WT | Amplified | Deletionc.267 | CDKN2A | >10 |
| Hey | Serous | G1391A | WT | WT | WT | WT | UN | 0.01 (0.001) |
| OVCAR8 | Serous | WT | WT | WT | G2327T | G376A | UN | >10 |
Mutation data were obtained from the catalog of somatic mutations in cancer database, COSMIC (http://www.sanger.ac.uk/genetics/CGP/cosmic/).
Histology data was obtained from ATCC and COSMIC.
IC50 corresponds to doses that result in 50% growth inhibition, relative to vehicle-only treated control cells.
Data are expressed as mean (±SEM), determined for a range of drug concentrations.
UN: unknown
WT: wild type
COSMIC database (Sanger.co.uk)
